# Transseptal coronary artery—a pictorial review

**DOI:** 10.1007/s00247-024-05911-x

**Published:** 2024-04-19

**Authors:** Vijetha V Maller, Jason N. Johnson, Umar Boston, Christopher Knott-Craig

**Affiliations:** 1 Department of Radiology, LeBonheur Children’s Hospital, University of Tennessee Health Science Center, 848 Adams Avenue, Radiology G216, Memphis, TN USA; 2 Division of Pediatric Cardiology, Pediatrics, Heart institute, LeBonheur Children’s Hospital, University of Tennessee Health Science Center, Memphis, TN USA; 3 Division of Pediatric Cardiothoracic Surgery, Department of Surgery, Heart Institute, LeBonheur Children’s Hospital, University of Tennessee Health Science Center, Memphis, TN USA

**Keywords:** Coronary artery, Interarterial, Intramural, Transconal, Transseptal, Unroofing, Virtual endoluminal

## Abstract

A transseptal coronary artery course, also known as a transconal course, is an anomalous course of the left main coronary artery (LMCA) or the left anterior descending artery (LAD) through the conal septal myocardium. The conal septal myocardium is the posterior wall of the right ventricular outflow tract (RVOT), acting as a dividing myocardial wall between the subaortic and subpulmonary outflow tracts. The initial segment of a transseptal coronary artery has an extraconal course between the aorta and the RVOT cranial to the true intramyocardial segment. The transseptal coronary artery then emerges out of the conal septal myocardium at the epicardial surface on the lateral aspect of the RVOT. Many consider the transseptal coronary artery to be a benign entity. However, there are few case reports of severe cardiac symptoms such as myocardial ischemia, arrhythmia, and even sudden cardiac deaths due to potential coronary artery compression in the systolic phase.​ In this article, we seek to describe the imaging findings of transseptal coronary artery course on coronary computed tomography angiography (CTA), discuss their clinical analysis, and briefly discuss the management of these lesions.

**Graphical abstract:**

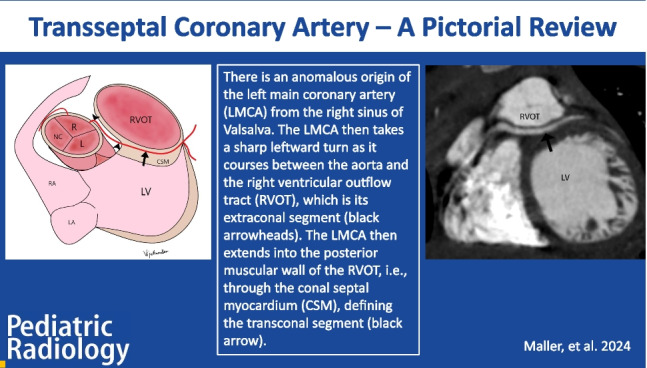

## Introduction

**Fig. 1:**
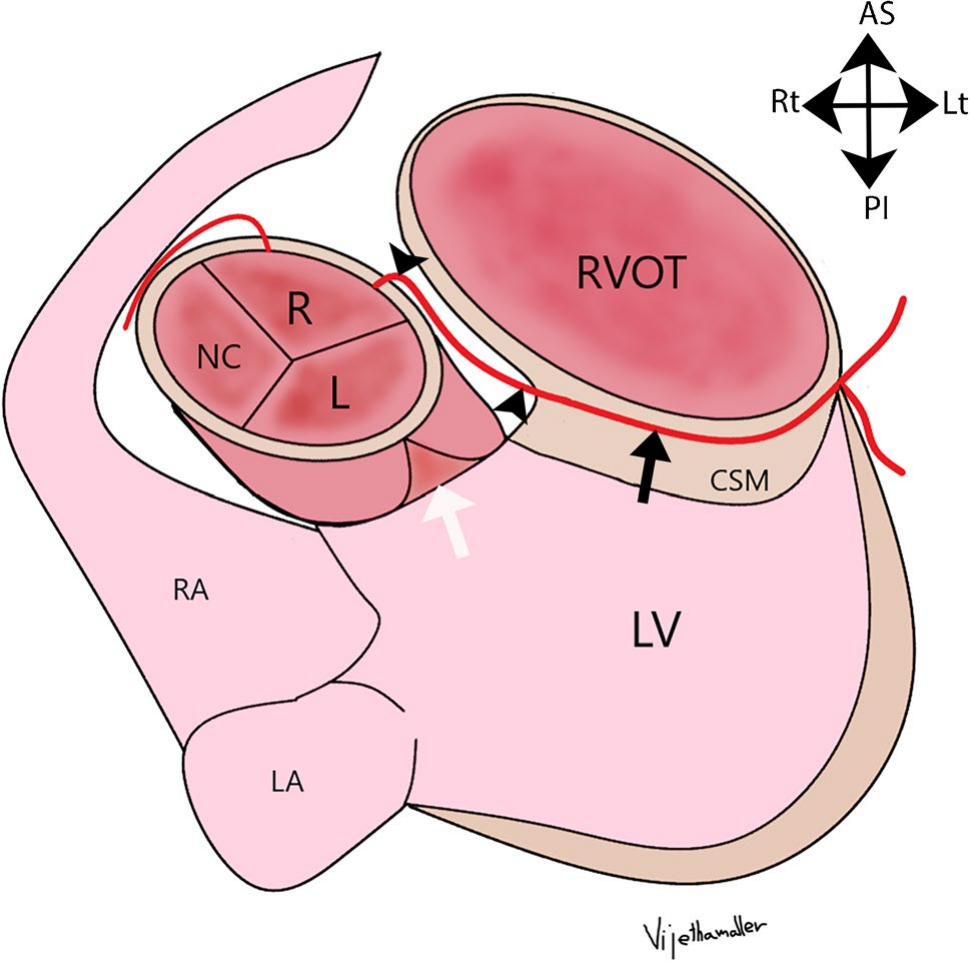
Illustration of transseptal course of anomalous aortic origin of left main coronary artery (LMCA). Diagram in an oblique axial plane. There is an anomalous origin of LMCA from the right sinus of Valsalva (R). The LMCA then takes a sharp leftward turn as it courses between the aorta and the RVOT, which is its extraconal segment (black arrowheads). The LMCA then extends below the level of the aortic annulus (white arrow) into the posterior muscular wall of the right ventricular outflow tract (RVOT), i.e., through the conal septal myocardium (CSM), defining the transconal segment (black arrow). The LMCA, after exiting out of the transconal segment, divides into LAD (left anterior descending artery) and LCx (left circumflex artery). The RCA originates normally from the right aortic sinus of Valsalva. LV, left ventricle; LA , left atrium; RA, right atrium; R, right coronary sinus; L, left coronary sinus; NC, non-coronary sinus; AS, anterior superior; PI, posterior inferior; Rt, right; Lt, left

Anomalous aortic origin of LMCA arising from the right coronary sinus of Valsalva is rare (0.02–0.1%); however, it carries a higher risk of sudden cardiac death when compared to anomalous aortic origin of right coronary artery (RCA) from the left coronary sinus of Valsalva [1]. When there is an anomalous aortic origin of LMCA or LAD directly from the right sinus of Valsalva or as a branch of the single coronary artery from the right sinus, the anomalous coronary artery (LMCA or LAD) may take an interarterial course (between the aorta and the pulmonary artery), pre-pulmonary course (anterior to pulmonary artery), or a transseptal course. The transseptal coronary artery is a rare variety of congenital coronary artery anomalies of LMCA or LAD. A transseptal course is defined as an anomalous course of anomalous aortic origin of LMCA or LAD through the conal septum after an initial extraconal course between the aorta and RVOT (Fig. 1). It is the transconal segment (and not the initial extraconal segment) of this anomalous LMCA or LAD, which is clinically relevant because this segment may be prone to systolic compression. Coronary CTA utilizing multiplanar, endoluminal, and three-dimensional (3D) reconstruction can help assess the true length of the transconal segment and determine the septal myocardial thickness overlying this segment. This can help the cardiac multidisciplinary team determine the appropriate management of such patients. While most patients remain asymptomatic, many symptomatic patients may be managed conservatively. There exist controversies regarding the surgical management of such patients since there are only a few case reports of myocardial ischemia and sudden cardiac deaths. Management of transseptal coronary artery course may be challenging due to the complexity of surgical interventions and the lack of available long-term follow-up data.​ Coronary CTA can provide presurgical analysis to assist in planning patients who may require surgical repair [2, 3]. Coronary CTA is essential to distinguish the anomalous origin of LMCA with transseptal course from interarterial and intramural course (Table 1) because the latter is usually treated by surgical unroofing or reimplantation and none of these surgeries would be appropriate for the transseptal course.

**Table 1:** Difference between transseptal, interarterial, and intramural courses

	Transseptal coronary artery	Interarterial coronary artery	Interarterial coronary artery with an initial intramural segment
Associations	It can only be seen in conjunction with the anomalous origin of the LMCA or LAD from the right coronary sinus or as a branch of the single coronary artery from the right sinus	It can be seen in conjunction with the anomalous origin of RCA, LMCA, or LAD from the opposite sinus or a branch of the single coronary artery from the opposite sinus	It can be in conjunction with anomalous aortic origin of RCA, LMCA, or LAD but not as a single coronary artery branch
Aortic origin	It has a right-angled or less acute angle of aortic origin and does not appear to abut the aorta after its origin	It has a less acute angle of aortic origin (> 45 degrees) and does not appear to abut the aorta after its origin	It has a hyperacute angle of aortic origin (< 45 degrees), with its course abutting the aorta until it exits the intramural segment
Ostium	The ostium is round	The ostium is round	The ostium is slitlike
Origin- relative to the pulmonary annulus	Its origin is below the level of the pulmonary annulus	Its origin is above the level of the pulmonary annulus	Its origin is above the level of the pulmonary annulus
Proximal course	After the anomalous origin, the initial (extraconal) segment courses between the aorta and RVOT	After the anomalous origin, the coronary artery courses between the aorta and pulmonary artery	After the anomalous origin, in addition to the interarterial course, the coronary artery courses within the aortic wall
Proximal course- luminal shape	The initial segment (extraconal segment) maintains a round caliber (assessed on the sagittal plane)	The interarterial segment may or may not have an elliptical luminal caliber (assessed on the sagittal plane)	The initial segment (intramural) always shows elliptical luminal caliber (assessed on sagittal plane)
Subsequent course	After the extraconal course, the transeptal segment dips below the aortic and pulmonary annulus levels into the conal septal myocardium	After completing the interarterial course, the coronary artery may extend below the pulmonary annulus before extending laterally in the atrioventricular groove. It does not dip down onto the septal myocardium below the level of the aortic annulus	After completing the interarterial intramural course, the coronary artery may extend below the pulmonary annulus before extending laterally in the atrioventricular groove. It does not dip down onto the septal myocardium below the level of the aortic annulus

## Imaging findings on coronary CTA

**Fig. 2:**
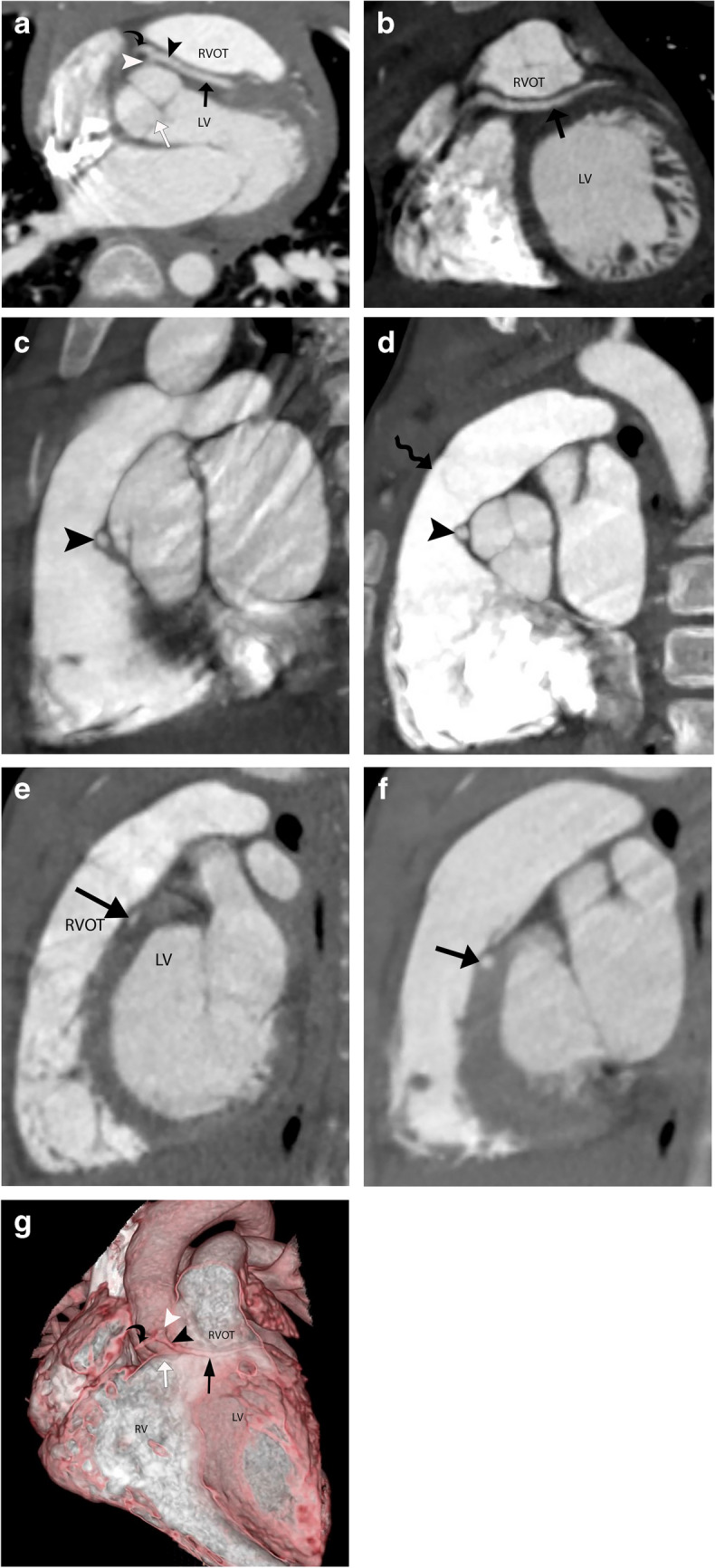
A 4-year-old asymptomatic male with a single coronary artery originating from the right sinus of Valsalva with a transseptal course of the left main coronary artery (LMCA). **a** Coronary CT angiography (CTA), oblique axial image. A single coronary artery (white arrowhead) originates from the right sinus of Valsalva, which then bifurcates into the right coronary artery (RCA) (black curved arrow) and LMCA (black arrowhead). The LMCA initially courses extraconally (black arrowhead) between the aorta and the right ventricular outflow tract (RVOT). The LMCA dips into the conal septum below the aortic annulus, defining the transseptal segment (black arrow). A white arrow points to the aortic annulus. **b** Coronary CTA, oblique coronal image. The LMCA (black arrow) courses through the conal septal myocardium with a hammock sign. LV, left ventricle. **c** Coronary CTA, oblique sagittal image in systolic phase (30% of RR interval). The LMCA (black arrowhead) courses extraconally between the distended aortic root and the contracted RVOT, maintaining a round caliber. **d** Coronary CTA, oblique sagittal image in diastolic phase (70% of RR interval). The LMCA (black arrowhead) courses extraconally between the aorta and the RVOT (below the level of pulmonary annulus shown by a wavy black arrow). The extraconal segment maintains a round caliber. **e** Coronary CTA oblique sagittal image in systolic phase (30% of RR interval). The LMCA (black arrow) courses through the septal myocardium between the RVOT and left ventricle (LV). The transconal LMCA (black arrow) shows an elliptical luminal caliber in the systolic phase, suggesting its myocardial compression in the systolic phase, unlike its extraconal segment. **f** Coronary CTA, oblique sagittal image in diastolic phase (70% of RR interval). The LMCA (black arrow) courses through the conal septal myocardium with a round luminal caliber. **g** 3D reformatted coronary CTA image, open view through an anterior approach. The LMCA (black arrowhead) originates from a single coronary artery (white arrowhead) arising from the right sinus of Valsalva. The extraconal segment of the LMCA (black arrowhead) extends from its origin to the level of the aortic annulus (white arrow ) between the aorta and RVOT. The intraconal segment is the hammock-like transverse segment (black arrow) below the level of the aortic annulus through the posterior wall of RVOT

**Fig. 3:**
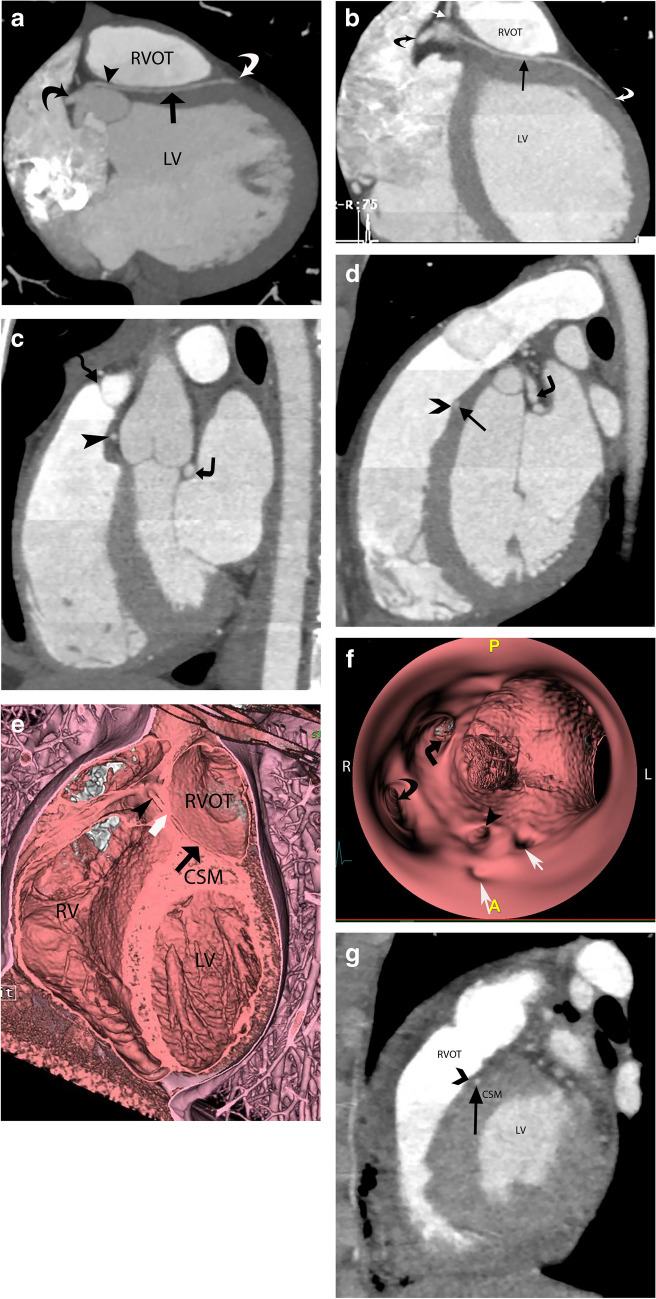
A 17-year-old male with a cardiac murmur and anomalous aortic origin of the left anterior descending artery (LAD) from the right sinus with a transseptal course. **a** Coronary CT angiography (CTA), oblique axial image. LAD has an anomalous origin from the right sinus of Valsalva, after which it takes a sharp turn towards the left, coursing between the aorta and right ventricular outflow tract (RVOT). A black arrowhead points to the extraconal segment of LAD. The LAD extends into the conal septal myocardium (black arrow), defining its transconal course, and then exits out at the epicardial surface (white curved arrow). The right coronary artery (RCA) (curved black arrow) has a normal origin. LV, left ventricle. **b** Coronary CTA, oblique coronal image. After its anomalous origin, the LAD extends into the conal septal myocardium, defining its transconal segment (black arrow) until it extends to the epicardial surface (curved white arrow). The RCA (curved black arrow) and conus artery (white arrow) originate from the right sinus of Valsalva. LV, left ventricle. **c** Coronary CTA, oblique sagittal image. The anomalously originating LAD (black arrowhead) courses below the level of the pulmonary annulus (wavy black arrow) between the aorta and RVOT in the extraconal space, maintaining its round luminal caliber. There is a benign retroaortic course of the LCX (right-angled black arrow). **d** Coronary CTA, oblique sagittal image in systolic phase (35% of RR interval). The LAD (black arrow) courses through the conal septal myocardium, with at least 1 mm overlying septal myocardium (black chevron). Due to myocardial compression, this segment’s luminal caliber is no longer round. The retroaortic course of the LCX is also noted (right-angled black arrow). There is a tangle of vessels associated with LCX due to coronary artery fistula with the pulmonary artery in this patient (not discussed here). **e** Coronary CTA, endoluminal view after virtual removal of the anterior aspect of the heart and anterior wall of RVOT. The black arrowhead shows an extraconal course of the anomalously originating LAD from the opposite sinus. The block white arrow points to the entry point of the LAD into the conal septal myocardium. The LAD follows a transconal course shown by the black arrows through the conal septal myocardium (CSM). RV, right ventricle. **f** Coronary CTA, endoluminal view of the aortic root obtained with the camera placed in the right sinus of Valsalva pointing towards the ostium of the anomalously originating LAD from the right sinus of Valsalva. The black arrowhead points to the ostium of LAD, originating within the right sinus of Valsalva. It has a round caliber with end-on visualization of its lumen without a slitlike ostium. Five ostia are located within the right sinus of Valsalva, including the LCx (right-angled black arrow), RCA (curved black arrow), LAD (black arrowhead), and two conus arteries (white arrows), in the order of right posterior to right anterior location. A, anterior; P,  posterior; R, right; L, left. **g** Coronary CTA after transconal unroofing of the LAD. Oblique sagittal image in systolic phase (30% of RR interval). The anomalously originating LAD (black arrow) courses posterior to the right ventricular outflow tract (RVOT). The LAD is anterior to the conal septal myocardium (CSM), covered anteriorly by the pericardial patch (black chevron) after transconal unroofing, and hence maintains a round luminal shape in the systolic phase compared to the preoperative imaging (Fig. 3d). LV, left ventricle

**Fig. 4:**
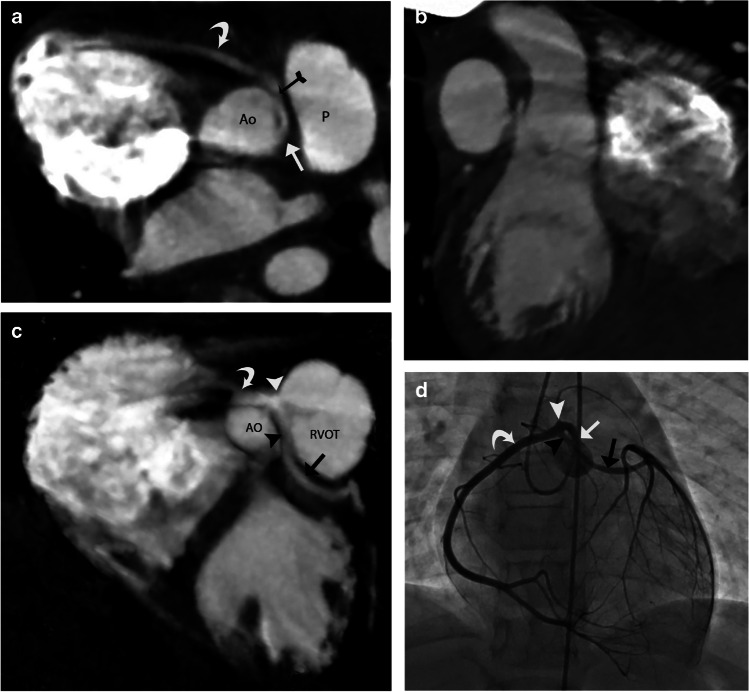
A 9-year-old male patient who presented with cardiac arrest was noted to have a single coronary artery arising from the left sinus of Valsalva with its interarterial intramural course and additional transseptal course of the left main coronary artery (LMCA). **a** Coronary CT angiography (CTA), oblique axial image. A single coronary artery arises from the left sinus of Valsalva (white arrow), followed by an interarterial course between the aorta (Ao) and pulmonary artery (P) with an intramural segment. It has a hyperacute angle of origin (white arrow) and a course along the aorta. The black-tailed arrow points to the exit point of the intramural segment. The curved white arrow points to the normal course of the right coronary artery (RCA), the branch of the anomalous single coronary artery. **b** Coronary CTA, oblique coronal image. An elliptical luminal shape (white arrow) of the interarterial intramural single coronary artery is noted, consistent with compression. **c** Coronary CTA, oblique coronal image. The white arrowhead points at the bifurcation of the single coronary artery after it exits out of the intramural segment at the anterior right sinus into the RCA (curved white arrow) and LMCA (black arrowhead). The LMCA extends between the aorta (Ao) and right ventricular outflow tract (RVOT), followed by the transconal course (black arrow) as it dips into the conal septal myocardium. **d** Catheter angiography frontal view with selective catheter injection of the single coronary artery at the left sinus of Valsalva. The single coronary artery (white arrow), after originating from the left sinus of Valsalva, extends towards the right side, indicating its interarterial course. The white arrowhead points to the bifurcation of the single coronary artery into RCA (curved white arrow) and LMCA (black arrowhead). The LMCA then dips down like a hammock at the expected level of the RVOT, consistent with its transseptal course (black arrow). The LMCA then branches out at the lateral margin of the heart

The transseptal course of the coronary artery is seen in conjunction with the anomalous origin of the LMCA (Fig. 2) or LAD (Fig. 3) from the right coronary sinus or as a branch of the single coronary artery from the right sinus. The angle of the aortic origin of a transseptal coronary artery is that of a right angle or less acute rather than a hyperacute angle of origin in an intramural coronary artery. On an axial plane or an oblique axial plane (Figs. 2a and 3a), the transseptal coronary artery, after its origin, immediately takes a sharp downward and leftward turn with the initial extraconal portion running between the aorta (above the aortic annulus) and RVOT, below the pulmonary annulus. It then defines its true transconal course as it traverses below the aortic and pulmonary annulus level through the posterior wall of RVOT. On oblique coronal view, the transconal segment takes a downward dip into the conal septal myocardium below the level of pulmonary annulus. This sign is called the “Hammock sign” because this downward dip of the transeptal coronary artery resembles a hammock (Fig. 2b and g, and 3b). The coronary artery then courses laterally towards the lateral aspect of the pulmonary conus to emerge out onto the epicardium. The “hammock sign” was initially described on conventional catheter angiography for the “downward dip” that a transseptal LMCA or LAD makes as it traverses below the level of the pulmonary valve in the septal myocardium [4] (Fig. 4d).

In the oblique sagittal CTA plane, the transseptal coronary artery originates below the level of the pulmonary annulus, differentiating it from the interarterial anomalous LMCA (which originates above the level of the pulmonary annulus). The initial segment maintains its round caliber (Figs. 2c and d, and 3c) due to the non-simultaneous distention of the aorta and RVOT. This helps differentiate it from the interarterial intramural LMCA, which has an elliptical luminal caliber due to its compression within the aortic wall and between the aorta and pulmonary artery. The transconal segment is surrounded by the septal myocardium (Figs. 2e and f, and 3d) on the sagittal plane. During the systolic phase (coronary CTA performed as a dose-modulated extended prospective or retrospective imaging), the transconal segment can have an elliptical luminal caliber (Figs. 2e and 3d) due to its potential compression by the surrounding myocardium.

The 3D reformats and endoluminal views (Fig. 3e and f) can define the length of the transseptal course and help evaluate the ostium, respectively. The ostium of a transseptal LMCA is round (Fig. 3f), contrary to the slitlike orifice seen with an interarterial intramural anomalous left coronary artery [5]. This is because the transseptal coronary artery has no common aortic media, unlike an interarterial intramural coronary artery. The location of the ostium of the transseptal LMCA is usually central within the right coronary sinus of Valsalva and not juxta-commissural. When the LMCA or LAD originates as a single coronary artery branch, there can never be a slitlike ostium or a proximal intramural course since the anomalous coronary artery does not originate directly from the aortic sinus.

Very rarely, both interarterial intramural and transseptal coronary artery anomalies may co-occur. For example, an anomalous single coronary artery originating from the left sinus with an initial interarterial intramural course exits out of the intramural course at the right sinus and bifurcates into RCA and LMCA. The LMCA then takes a transseptal course (Fig. 4).

It is essential to know that, unlike the anomalous aortic origin of LMCA, the anomalously originating RCA can never take a transseptal course even though it may take a course between the aorta and RVOT (described by some as a low interarterial course but not a true interarterial course), considered a benign variant unless it has an associated intramural segment [6].

Another point to note is that a transseptal coronary artery has physiological similarities with myocardial bridging. Myocardial bridging is coronary artery tunneling (LAD, LMCA, or RCA) under the left ventricular or right ventricular myocardium (not the conal septum). It is usually not associated with an anomalous aortic origin of the coronary artery. The thickness of the overlying myocardium in myocardial bridging may vary from 1 to 10 mm [7]. The thickness of the overlying septal myocardium with the transseptal coronary artery is usually not more than 1 mm [7].

### Clinical significance

Differentiating a transseptal coronary artery from the interarterial intramural LMCA is critical since the latter have a high risk of sudden cardiac death and are always surgically treated.

Most patients with transseptal coronary artery are asymptomatic. However, there can be a potential systolic compression with a milking effect on its transconal actional segment by the surrounding conal septal myocardium [8].

Based on a study by Doan et al., in a series of 18 patients (ages ranging from 3 months to 16 years) with transseptal anomalous left coronary artery, only 4 patients had exertional symptoms with associated inducible myocardial ischemia. Only 30% of asymptomatic patients had inducible myocardial ischemia. Surgical management with coronary artery bypass grafting was performed in one patient, whereas the rest with inducible myocardial hypoperfusion and impaired coronary flow were managed conservatively [8]. Based on a literature review conducted by Glushko et al. in 74 reported cases of the transseptal coronary artery, 26% were symptomatic, 11% had sudden cardiac death, 11% presented with myocardial ischemia, angina, or reported chest pain, 2% had palpitations or exercise-induced neuro-cardiogenic spells which improved after cardiac bypass, and 1% had persistent ventricular tachycardia [7].

### Management

There are no consensus guidelines for managing transseptal coronary artery anomaly, as it is a rare diagnosis with variable symptoms and risks. The current strategy involves assessing patient symptoms, confirming coronary artery anatomy, and evaluating for evidence of myocardial ischemia or infarction [9]. The methods that the centers use to evaluate ischemia are based on practice patterns and expertise within institutions.

**Fig. 5:**
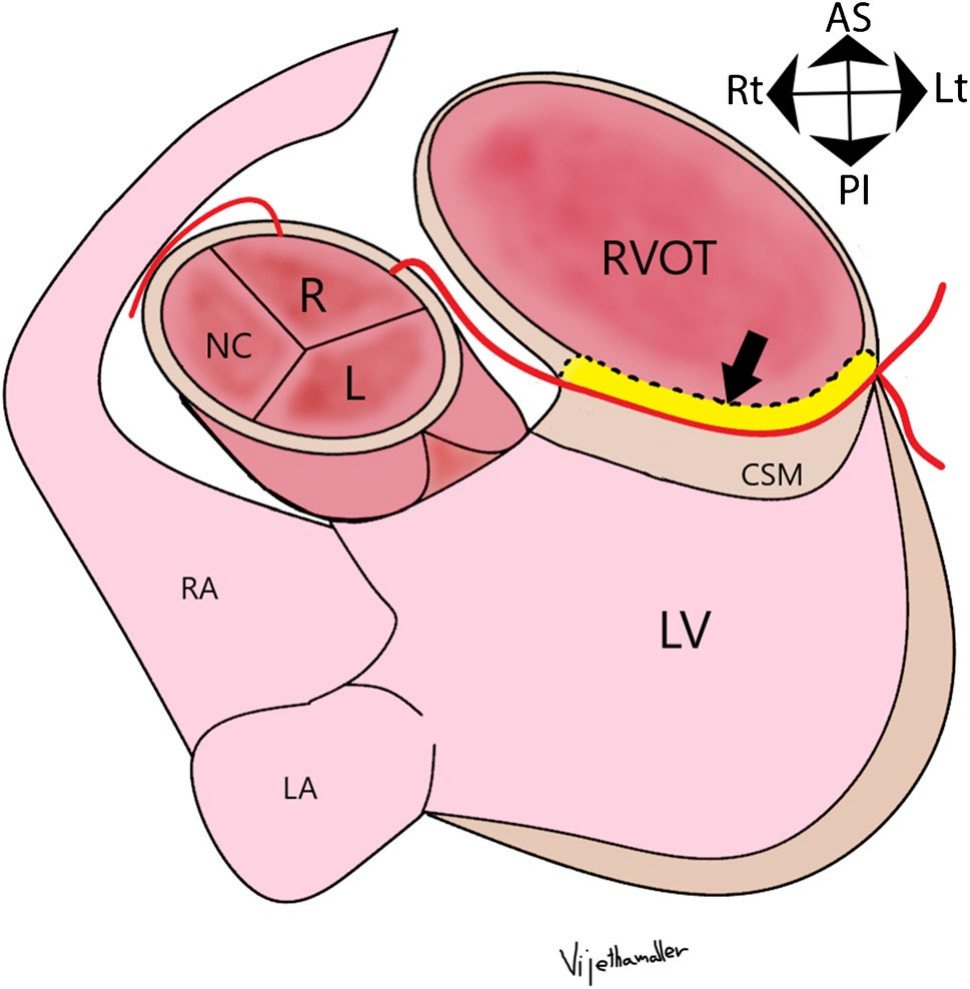
Illustration of transconal unroofing of left main coronary artery (LMCA) in an oblique axial plane. The conal septal myocardium overlying the LMCA is excised (compared to Fig. 1), and the posterior wall of the right ventricular outflow tract (RVOT) is repaired with a rectangular patch of fresh autologous pericardial patch. The block black arrow points to the yellow color-coded autologous pericardium, replacing the excised portion of the septal myocardium. LV, left ventricle; LA, left atrium; RA, right atrium; R, right coronary sinus; L, left coronary sinus; NC, non-coronary sinus; AS, anterior superior; PI, posterior inferior; Rt, right; Lt, left

An expectant management approach would be reasonable in asymptomatic patients with no evidence of ischemia. In asymptomatic patients but with evidence of ischemia, the management is controversial. Usually, a shared decision-making approach is adopted in these cases after consultation with cardiology and cardiovascular surgery to understand the risks and benefits of both options. Surgical repair would be indicated to relieve the ischemia in patients with cardiovascular symptoms and evidence of ischemia [10]. Coronary artery bypass graft (CABG) or mobilization of the pulmonary root and incising the overlying muscle bridge with translocation of the right pulmonary artery are known surgical techniques for the transseptal course [3, 11]. A newer surgical technique with a transconal approach includes transection of the RVOT, unroofing the septal course of the LMCA or LAD, followed by repair of the posterior wall of RVOT with autologous pericardial patch [2, 3] (Figs. 3g and 5).

### Summary

An anomalous aortic origin of LMCA or LAD with a transseptal course is a rare congenital coronary artery anomaly that is considered by many as a benign anomaly that can be conservatively managed. However, it can sometimes be clinically significant, requiring surgical intervention. Cardiac imagers should be familiar with the imaging appearance of the transseptal coronary artery and how to differentiate it from other anomalies of coronary origin and course.
